# Strong and Ductile Non-equiatomic High-Entropy Alloys: Design, Processing, Microstructure, and Mechanical Properties

**DOI:** 10.1007/s11837-017-2540-2

**Published:** 2017-08-21

**Authors:** Zhiming Li, Dierk Raabe

**Affiliations:** Max-Planck-Institut für Eisenforschung, Max-Planck-Str. 1, 40237 Düsseldorf, Germany

## Abstract

We present a brief overview on recent developments in the field of strong and ductile non-equiatomic high-entropy alloys (HEAs). The materials reviewed are mainly based on massive transition-metal solute solutions and exhibit a broad spectrum of microstructures and mechanical properties. Three relevant aspects of such non-equiatomic HEAs with excellent strength–ductility combination are addressed in detail, namely phase stability-guided design, controlled and inexpensive bulk metallurgical processing routes for appropriate microstructure and compositional homogeneity, and the resultant microstructure–property relations. In addition to the multiple principal substitutional elements used in these alloys, minor interstitial alloying elements are also considered. We show that various groups of strong and ductile HEAs can be obtained by shifting the alloy design strategy from single-phase equiatomic to dual- or multiphase non-equiatomic compositional configurations with carefully designed phase instability. This design direction provides ample possibilities for joint activation of a number of strengthening and toughening mechanisms. Some potential research efforts which can be conducted in the future are also proposed.

## Introduction

Conventional alloy design over the past centuries has been constrained by the concept of one or two prevalent base elements. As a breakthrough of this restriction, the concept of high-entropy alloys (HEAs) containing multiple principal elements has drawn great attention over the last 13 years due to the numerous opportunities for investigations in the huge unexplored compositional space of multicomponent alloys.^1^–^6^ A large number of studies in this field have been motivated by the original HEA concept, which suggested that achieving maximized configurational entropy using equiatomic ratios of multiple principal elements could stabilize single-phase massive solid-solution phases.^1^

However, an increasing number of studies have revealed that formation of single-phase solid solutions in HEAs shows weak dependence on maximization of the configurational entropy through equiatomic ratios of elements,^7^–^10^ and it was even found that maximum entropy is not the most essential parameter when designing multicomponent alloys with superior properties.^11^,^12^ These findings encouraged efforts to relax the unnecessary restrictions on both the equiatomic ratio of multiple principal elements as well as the formation of single-phase solid solutions. In this context, non-equiatomic HEAs with single-, dual-, or multiphase structure have recently been proposed to explore the flexibility of HEA design and overcome the limitations of the original HEA design concept.^4^,^13^,^14^ Also, deviation from the equimolar composition rule facilitates identification of compositions which allow the often-brittle intermetallic phases to be avoided.

**Fig. 1:**
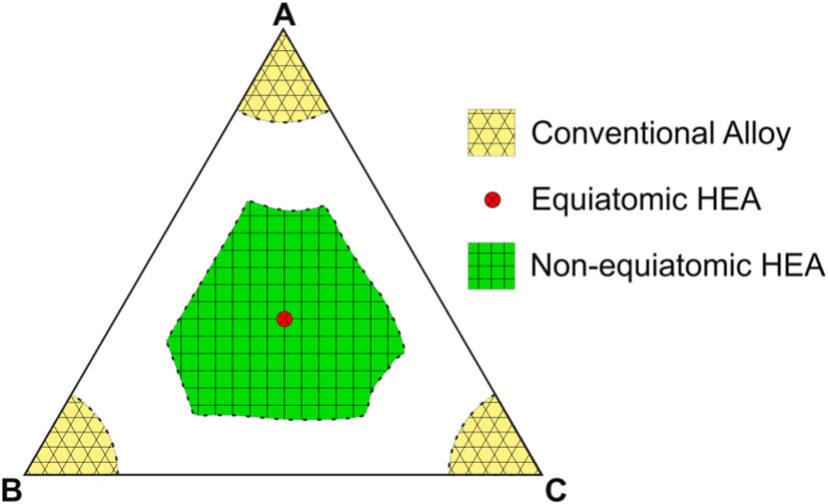
Schematic diagram showing the compositional space of non-equiatomic high-entropy alloys (HEAs), which is significantly larger than that of conventional alloys or equiatomic HEAs

Thermodynamic investigations of non-equiatomic HEAs showed that the configurational entropy curve of these alloys is rather flat, indicating that a wide range of compositions alongside the equiatomic configuration assume similar entropy values.^15^ As illustrated schematically in Fig. 1, compared with conventional alloys with one or two principal elements plus minor alloying components, as well as equiatomic HEAs with equimolar ratios of all alloy elements, non-equiatomic HEAs greatly expand the compositional space that can be probed. Indeed, recent studies have revealed that outstanding mechanical properties exceeding those of equiatomic HEAs can be achieved by non-equiatomic alloys.^4^,^13^ As one of the possible pathways, a novel type of transformation-induced plasticity-assisted dual-phase (TRIP-DP) HEA was developed.^4^ The two constituent phases in the alloy, i.e., the face-centered cubic (FCC) matrix and the hexagonal close-packed (HCP) phase, are compositionally equivalent and thus can both be referred to as high-entropy phases.^4^ This leads to a significantly improved strength–ductility combination compared with corresponding equiatomic HEAs, mainly due to the combination of massive solid-solution strengthening and the TRIP effect.^4^,^13^ The above-mentioned findings clearly indicate that expanding the HEA design concept to non-equiatomic compositions has great potential for pursuing more compositional opportunities for design of novel materials with exceptional properties.

We aim herein to provide a brief overview on some recent developments of various strong and ductile non-equiatomic HEAs, placing specific attention on their compositional design, metallurgical processing routes, and microstructure–property relations. Some pending directions in this field, e.g., multifunctionalities of various non-equiatomic HEAs, are also pointed out.

## Compositional Design of Strong and Ductile Non-equiatomic High-Entropy Alloys

Since the majority of the research effort in the field of HEAs during the last decade has focused on single-phase solid solutions, compositional design criteria for achieving single-phase solid solutions have been well explored; For instance, it was proposed that valence electron concentration (VEC) is a critical parameter determining the stability of single-phase FCC (VEC ≥ 8) and body-centered cubic (BCC) (VEC < 6.87) solid solutions.^16^ However, the limited hardening mechanisms available in single-phase HEAs, i.e., primarily dislocation interaction and solid-solution strengthening, restrict their strain-hardening capacity as well as the attainable strength–ductility combination.

Using the concept of non-equiatomic HEAs, one can tune the compositions of certain HEA systems to introduce multiple deformation mechanisms, thereby improving the range of strength–ductility combinations accessible. This is attributed to the fact that high ductility of strong metallic alloys can be obtained when different deformation mechanisms are activated sequentially during ongoing loading, such as the additional activation of twinning and phase transformation at higher deformations known from twinning-induced plasticity (TWIP) and TRIP steels.^17^,^18^ The TWIP and TRIP phenomena are mainly determined by the value of the stacking fault energy, i.e., the energy carried by the interruption of the normal stacking sequence.^19^–^21^ The intrinsic stacking fault energy $\gamma_{\text{I}}$ of FCC-structured alloys can be expressed as^22^

1 $$ \gamma_{\text{I}} = 2\rho_{\text{A}}\Delta G^{{{\text{FCC}} \to {\text{HCP}}}} + 2\sigma^{{{\text{FCC}} \to {\text{HCP}}}} , $$

where $\Delta G^{{{\text{FCC}} \to {\text{HCP}}}}$ is the molar free energy difference between the FCC and HCP phase, $\rho_{\text{A}}$ is the planar packing density (moles/area) of a close-packed plane, and $\sigma^{{{\text{FCC}} \to {\text{HCP}}}}$ is the coherent FCC-HCP interfacial energy. For transition metals, the interfacial energy has been assumed to be 10 ± 5 mJ/m^2^.^20^,^22^ The intrinsic stacking fault energy is then mainly determined by the free energy difference between the FCC and HCP phases for compositions of a certain alloy system with similar planar packing density of the close-packed planes. Therefore, both phenomena, i.e., TWIP and TRIP, can be best tuned not by achieving the highest possible entropy-driven thermodynamic stability of the host FCC-structured solid solution but instead by its phase instability related to the free energy difference between the phases, rendering it amenable to athermal transformation mechanisms.

**Fig. 2:**
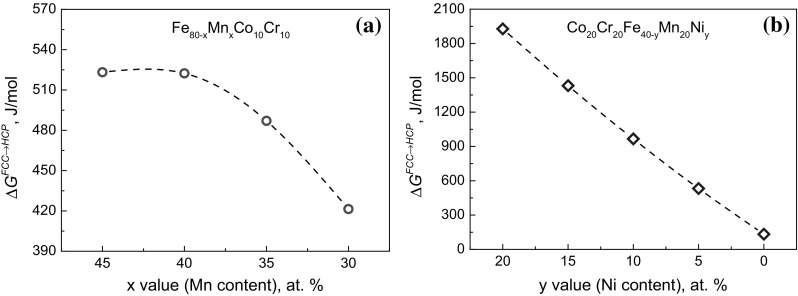
Free energy differences (Δ*G*) between the FCC and HCP structures of typical alloy systems at 300 K derived by thermodynamic calculations using the Calphad approach (Thermo-Calc, database TCFE7): (a) quaternary Fe_80−*x*_Mn_*x*_Co_10_Cr_10_ (*x* = 45 at.%, 40 at.%, 35 at.%, and 30 at.%) and (b) quinary Co_20_Cr_20_Fe_40−*y*_Mn_20_Ni_*y*_ (*y* = 20 at.%, 15 at.%, 10, 5 at.%, and 0 at.%)

Figure 2 shows the free energy differences between the FCC and HCP structures of two typical alloy systems, i.e., quaternary Fe_80−*x*_Mn_*x*_Co_10_Cr_10_ (*x* = 45 at.%, 40 at.%, 35 at.%, and 30 at.%) and quinary Co_20_Cr_20_Fe_40−*y*_Mn_20_Ni_*y*_ (*y* = 20 at.%, 15 at.%, 10 at.%, 5 at.%, and 0 at.%), at 300 K derived by thermodynamic calculations using the Calphad approach. Note that parameter-free ab initio simulations, in particular density functional theory calculations, are also powerful to obtain stacking fault energies and/or free energy differences between phases.^23^ In the quaternary Fe_80−*x*_Mn_*x*_Co_10_Cr_10_ system, $\Delta G^{{{\text{FCC}} \to {\text{HCP}}}}$ decreases with reduction of the Mn content (*x* value) from 45 at.% to 30 at.% (Fig. 2a). Indeed, according to our experimental results,^4^ the Mn content plays an important role in the phase constitution and phase stability of this alloy system, enabling tuning of displacing transformation mechanisms, i.e., TWIP and TRIP. Specifically, the Fe_35_Mn_45_Co_10_Cr_10_ alloy has a single FCC structure with dislocation slip as the main deformation mechanism, while Fe_40_Mn_40_Co_10_Cr_10_ additionally exhibits a TWIP effect of the single FCC structure.^9^,^12^ With further decrease of the Mn content, which results in a decrease of $\Delta G^{{{\text{FCC}} \to {\text{HCP}}}}$ and hence of the FCC phase stability, the Fe_50_Mn_30_Co_10_Cr_10_ alloy exhibits partial martensitic transformation of the FCC to the HCP phase upon cooling from the high-temperature single-phase region,^4^,^13^ with the formation of a dual-phase HEA composed of two phases of identical chemical composition, i.e., high-entropy phases. This enables significant enhancement of both the strength and ductility of the quaternary Fe_80−*x*_Mn_*x*_Co_10_Cr_10_ system.^4^ Analogously, $\Delta G^{{{\text{FCC}} \to {\text{HCP}}}}$ decreases significantly with reduction of the Ni content (*y* value) from 20 at.% to 0 at.% in the quinary Co_20_Cr_20_Fe_40−*y*_Mn_20_Ni_*y*_ system (Fig. 2b). According to recent experimental results,^23^ the structure of the alloy accordingly shifts from single FCC phase to dual phase (FCC and HCP) at the homogenized state without strain loading when decreasing the Ni content in the Co_20_Cr_20_Fe_40−y_Mn_20_Ni_y_ system from 20 at.% to 6 at.%. Except for the dual-phase structure, the resultant non-equiatomic Co_20_Cr_20_Fe_34_Mn_20_Ni_6_ alloy also exhibits the TRIP effect and hence higher tensile strength and strain-hardening ability compared with the corresponding equiatomic reference Co_20_Cr_20_Fe_20_Mn_20_Ni_20_ alloy.^23^ This indicates that the TRIP-DP effect introduced into the former quaternary alloy can also be realized in quinary alloys with higher mixing entropy value.

When designing the composition of strong and ductile non-equiatomic dual- or multiphase HEAs, it is also essential to note that the multiple principal elements selected should be distributed uniformly in the microstructure, or at least partition in such a way that all of the coexisting phases have a high solid-solution effect and high mixing entropy. This was not achieved in previous studies, where dual- or multiphase structures were rather formed by elemental segregation, generally leading to undesired brittle intermetallic compounds.^3^ From this point of view, deformation-driven partial athermal martensitic transformation without associated chemical gradients across phase boundaries is likely to be a well-suited approach for producing dual- or multiphase HEAs with uniformly distributed multiple principal elements. The TRIP-DP effect explained above for addressing the strength–ductility trade-off can also be introduced into other types of HEAs such as the refractory metal TiNbTaZrHf system via a “*d*-electron alloy design” approach where athermal transitions between the BCC and the HCP phases are conceivable.^24^

Furthermore, minor interstitial element fractions can also be introduced into strong and ductile non-equiatomic dual- or multiphase HEAs to further improve their mechanical properties. We added carbon as interstitial element into a TRIP-DP-HEA in the pursuit of two main trends^25^: (i) that addition of interstitial carbon leads to a slight increase in stacking fault energy and hence phase stability, enabling tuning of the FCC matrix phase stability to a critical point so as to trigger the TWIP effect while maintaining the TRIP effect, thereby further improving the alloy’s strain-hardening ability; (ii) that HEAs can benefit profoundly from interstitial solid-solution strengthening with its huge local distortions instead of only the established massive substitutional solid-solution strengthening provided by its multiple principal elements. Thus-prepared interstitial HEA (referred to as iHEA) was indeed characterized by a combination of various strengthening mechanisms including interstitial and substitutional solid solution, TWIP, TRIP, nanoprecipitates, dislocation interactions, stacking faults, and grain boundaries, leading to twice the tensile strength compared with the equiatomic Co_20_Cr_20_Fe_20_Mn_20_Ni_20_ reference HEA while maintaining identical ductility.^25^

## Processing of Strong and Ductile Bulk Non-equiatomic High-Entropy Alloys

**Fig. 3:**
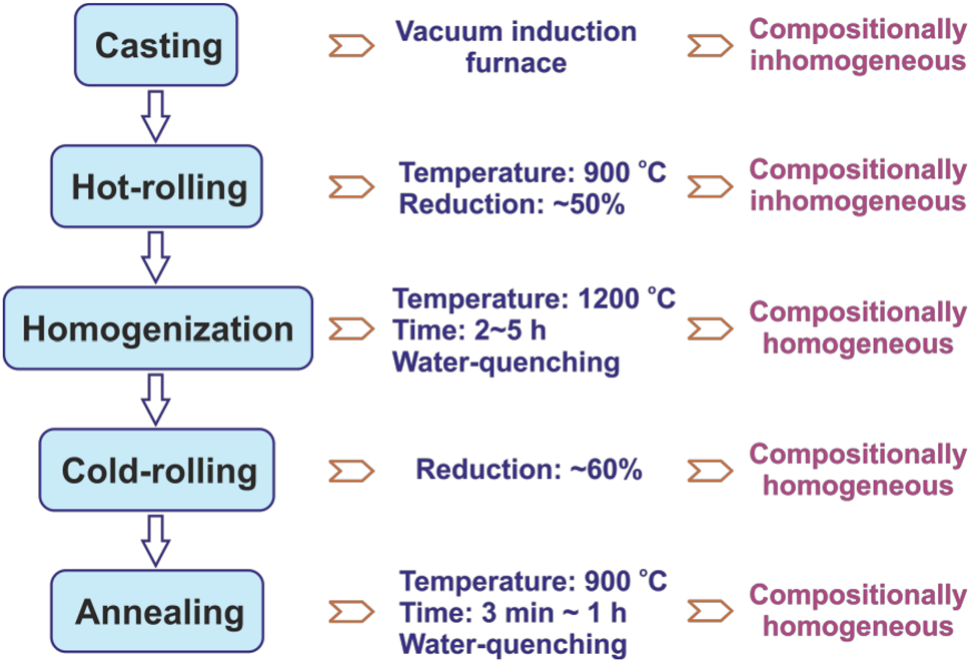
Processing routes and related parameters as well as resultant compositional homogeneity states for 3*d* transition-metal high-entropy alloys

For 3*d* transition-metal HEAs, well-established bulk metallurgical processes are available to synthesize high-quality alloy sheets when the thermomechanical processing parameters are controlled in a proper way. As shown in Fig. 3, we used a vacuum induction furnace to melt and cast various transition-metal HEAs. Except for traditional casting setups, the recently developed combinatorial approach, referred to as rapid alloy prototyping (RAP),^26^ can also be employed for rapid trend screening of suitable alloy compositions. The RAP technique enables synthesis of five different alloys with tuned compositions of the alloy system in one operation by using a set of five copper molds which can be moved stepwise inside the furnace.^26^

In as-cast condition, the multiple principal elements are typically not homogeneously distributed in the bulk HEAs with their coarse dendritic microstructure owing to classical Scheil segregation, although x-ray diffraction (XRD) analysis may suggest single- or dual-phase structures.^27^ Following casting, alloy plates are cut from the cast blocks and hot-rolled at 900°C with total thickness reduction of 50% to remove the dendritic microstructure and possible inherited casting defects. The hot-rolling temperature can be adjusted to higher values depending on the specific alloy compositions. Often, even such hot-rolled HEAs still show some retained compositional inhomogeneity. The hot-rolled alloy sheets are thus homogenized at 1200°C for more than 2 h followed by water quenching. The homogenization time should be extended according to the dimensions of the alloy sheets; i.e., the larger the alloy sheet, the longer the homogenization time. Such homogenized HEA sheets generally show homogeneous distribution of the multiple principal elements and no cracks or pores. However, for HEAs containing high amount of Mn (e.g., >10 at.%), there would be a few inclusions enriched in Mn, which are very hard to remove even by long-term homogenization.

Since homogenized HEA sheets exhibit huge grain size (>30 *μ*m), cold-rolling and annealing processes are generally required to refine the grains to achieve better mechanical properties. We cold-rolled homogenized alloy sheets to total thickness reduction of ~60% and annealed them at 900°C for different time periods. Annealing was conducted to obtain full recrystallization of the microstructure and to control the grain sizes, hence times and temperatures were adjusted in each case according to the targeted grain sizes and textures. Long-term annealing treatments (e.g., 500 days) at intermediate temperatures (e.g., 700°C and 500°C) can lead to chemical segregations even for the single-phase equiatomic CoCrFeMnNi HEA.^8^

**Fig. 4:**
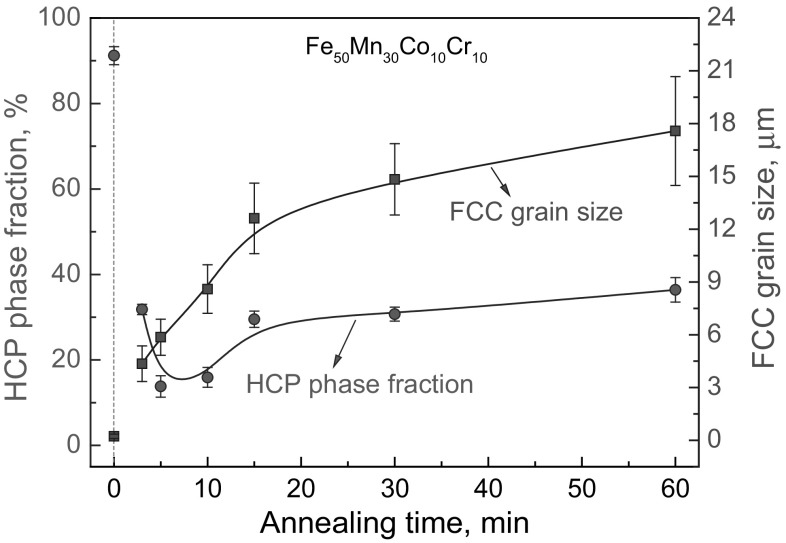
Variations in FCC grain size and HCP phase fraction in dual-phase Fe_50_Mn_30_Co_10_Cr_10_ alloy with increasing annealing time at 900°C. Annealing time of 0 min refers to the cold-rolled state of the samples without annealing. Data mainly taken from Ref. 13

Interestingly, for the recently designed TRIP-assisted dual-phase HEAs,^13^ annealing treatments can be used not only to control the grain size, but also to modify the phase fractions in the microstructure. Figure 4 shows the variations in the FCC grain size and HCP phase fraction of the quaternary dual-phase Fe_50_Mn_30_Co_10_Cr_10_ HEA with increasing annealing time at 900°C.^13^ In cold-rolled condition, the alloy shows average grain size of ~250 nm and HCP phase fraction of ~91%. After extending the annealing time from 3 min to 60 min, the average FCC grain size increased gradually from ~4.5 *μ*m to ~17.5 *μ*m. The average HCP phase fraction first drops to ~14% from ~32% when increasing the annealing time from 3 min to 5 min, then gradually increases to ~36% when further increasing the annealing time to 60 min. The underlying mechanisms responsible for this nonmonotonic variation of the HCP phase fraction with increasing annealing time and grain size are discussed in Ref. 13 by considering kinetic factors such as nucleation of the HCP phase and blocking effects of grain boundaries. Grain size and phase fractions significantly influence the deformation behavior of dual-phase HEAs, suggesting that a well-tuned annealing process is pivotal for achieving desired excellent strength–ductility combinations in these alloys.

Processing routes are not only decisive for the grain size and phase fraction corresponding to a targeted specific composition, but are also critical with respect to the compositional homogeneity state, which also has significant effects on the mechanical behavior. We applied various processing routes including hot-rolling, homogenization, cold-rolling, and recrystallization annealing on cast alloys to obtain samples in different compositional homogeneity states.^27^ In the case of coarse grains (~300 *µ*m) obtained for as-cast alloys without homogenization treatment, the ductility and strain hardening of the material were significantly reduced due to the compositional inhomogeneity. This detrimental effect was attributed to preferred deformation-driven phase transformation occurring in the Fe-enriched regions with lower stacking fault energy, promoting early stress–strain localization.^27^ The grain-refined structure (~4 *µ*m) with compositional heterogeneity obtained for annealed alloys without any preceding homogenization treatment was characterized by almost total loss of work hardening. This effect was attributed to large local shear strains due to inhomogeneous planar slip.^27^ These findings demonstrate the importance of processing routes for the development of strong and ductile HEAs.

## Microstructure and Mechanical Properties of Non-equiatomic High-Entropy Alloys

**Fig. 5:**
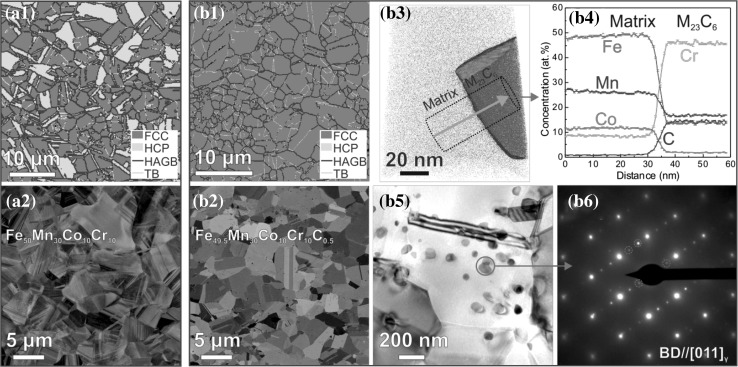
Typical microstructures of Fe_50_Mn_30_Co_10_Cr_10_ and Fe_49.5_Mn_30_Co_10_Cr_10_C_0.5_ alloys after recrystallization annealing for 3 min: (a_1_) EBSD phase map and (a_2_) ECC image of dual-phase Fe_50_Mn_30_Co_10_Cr_10_ alloy; (b_1_) EBSD phase map, (b_2_) ECC image, (b_3_) APT tip reconstruction, (b_4_) elemental profiles across an interface of matrix and carbide, (b_5_) TEM bright-field image, and (b_6_) selected-area diffraction pattern of interstitial Fe_49.5_Mn_30_Co_10_Cr_10_C_0.5_ alloy. Diffraction spots marked by red circles in (b_6_) show the FCC structure of the M_23_C_6_ carbides (Color figure online)

Similar to the single-phase equiatomic Co_20_Cr_20_Fe_20_Mn_20_Ni_20_ alloy, the single-phase non-equiatomic Fe_40_Mn_27_Ni_26_Co_5_Cr_2_,^11^ Fe_35_Mn_45_Co_10_Cr_10_,^9^ and Fe_40_Mn_40_Co_10_Cr_10_ 12 alloys can also form a fully recrystallized microstructure containing a large amount of annealing twins in equiaxed matrix grains with uniformly distributed elements when using appropriate processing routes as explained above. When shifting the single-phase non-equiatomic HEAs to dual-phase non-equiatomic HEAs and further to interstitially alloyed HEAs (iHEAs), the complexity of the microstructure gradually increases. Figure 5 shows the typical microstructures of the non-equiatomic Fe_50_Mn_30_Co_10_Cr_10_ TRIP-DP-HEA^4^,^13^ and Fe_49.5_Mn_30_Co_10_Cr_10_C_0.5_ TRIP-TWIP-iHEA^25^ as revealed by electron backscatter diffraction (EBSD), electron channeling contrast imaging (ECCI), atom probe tomography (APT), and transmission electron microscopy (TEM) techniques. The TRIP-DP-HEA consists of two phases, namely FCC γ matrix and HCP ε phase (Fig. 5a_1_). The HCP ε phase is formed within the FCC γ matrix and mainly exhibits laminate morphology (Fig. 5a_1–2_). Annealing twins, stacking faults, and dislocations are also present in the FCC γ matrix in recrystallized state.^4^,^13^ With addition of C, the fraction of HCP ε phase in the iHEA is significantly reduced after annealing (Fig. 5b_1_) compared with the reference alloy without C (Fig. 5a_1_). This is due to the slight increase of stacking fault energy and correspondingly higher FCC phase stability with addition of C. A large amount of annealing twins can also be observed in the iHEA in annealed state (Fig. 5b_1_). Also, particles with average size of 50–100 nm and volume fraction of ~1.5 vol.% are observed (Fig. 5b_2–6_). These particles were determined to be M_23_C_6_ carbides (M: Cr, Mn, Fe, and Co) with FCC structure according to APT (Fig. 5b_3–4_) and TEM (Fig. 5b_5–6_) probing.

**Fig. 6:**
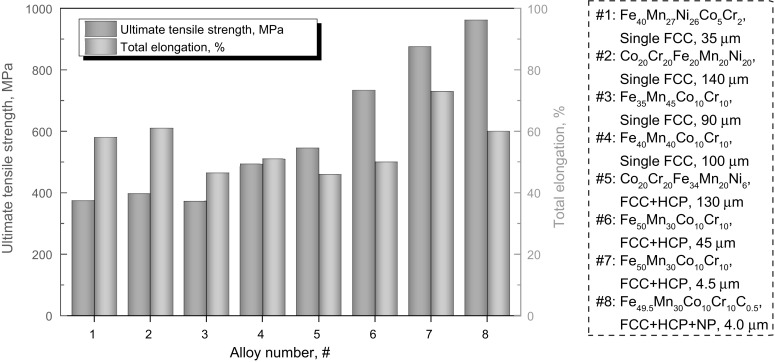
Overview of ultimate tensile strength and total engineering elongation obtained for various non-equiatomic high-entropy alloys. For comparison, data of the equiatomic Co_20_Cr_20_Fe_20_Mn_20_Ni_20_ alloy (#2) are also shown. All alloys produced in-house using similar processing routes shown in Fig. 2 for full control of the experimental setup. All these data stem from uniaxial tensile tests conducted on bulk samples with identical dimensions at room temperature at strain rate of 1 × 10^−3^ s^−1^

**Fig. 7:**
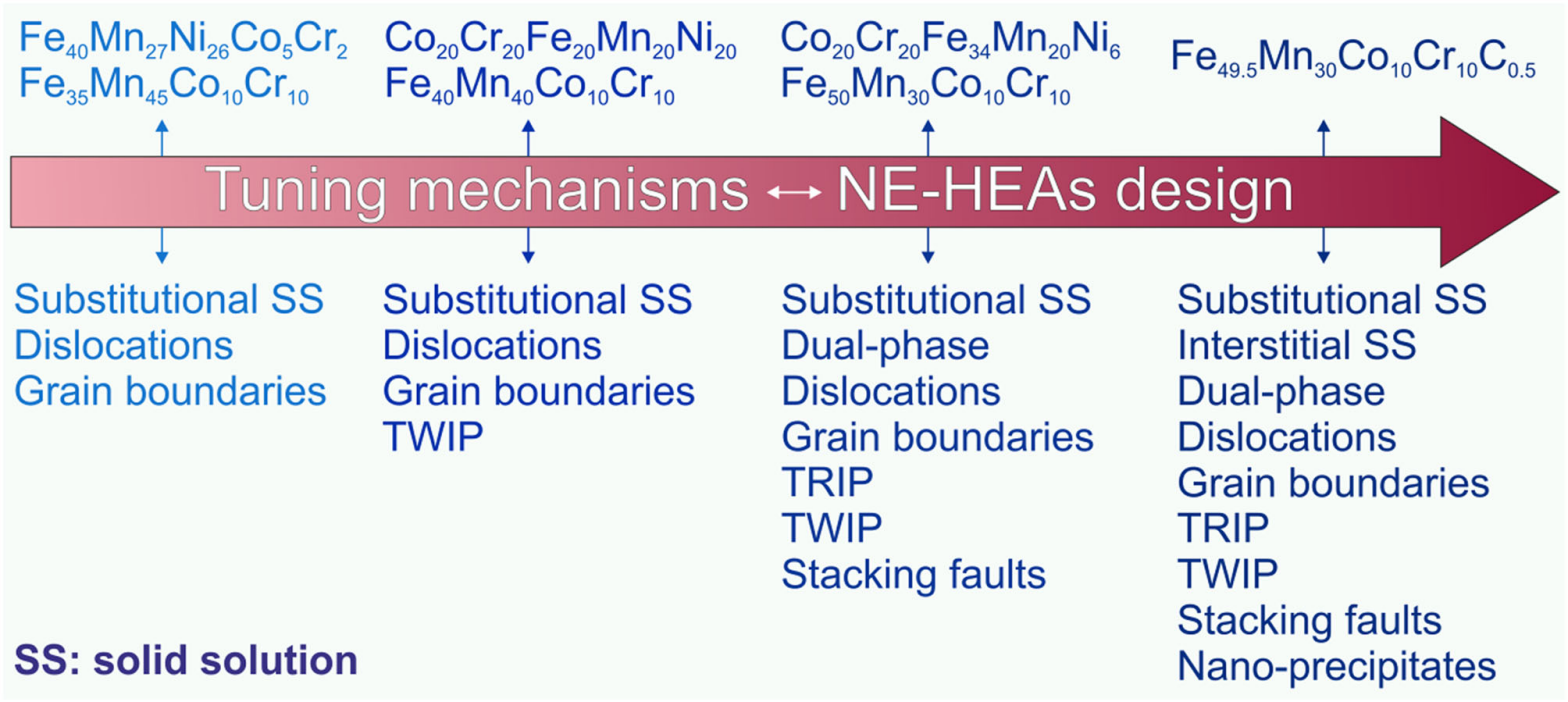
Overview of deformation mechanisms in various multicomponent high-entropy alloys showing that tuning deformation mechanisms is key to development of strong and ductile non-equiatomic high-entropy alloys (NE-HEAs). The strength and ductility of these alloys are given in Fig. 6. SS: solid solution

Figure 6 summarizes the ultimate tensile strength and total elongation data obtained from various transition-metal HEAs. The homogenized single-phase non-equiatomic Fe_40_Mn_27_Ni_26_Co_5_Cr_2_ (#1)^11^ alloy shows ultimate strength and total elongation values similar to the homogenized single-phase equiatomic Co_20_Cr_20_Fe_20_Mn_20_Ni_20_ alloy (#2),^23^ even though the former has finer grain size (35 versus 140 *μ*m; Fig. 7). The trend is similar for the homogenized non-equiatomic Fe_35_Mn_45_Co_10_Cr_10_ alloy (#3). One of the key mechanisms behind this trend is that twinning can occur in the single-phase equiatomic Co_20_Cr_20_Fe_20_Mn_20_Ni_20_ alloy to a certain extent, while mere dislocation hardening prevails in the non-equiatomic Fe_40_Mn_27_Ni_26_Co_5_Cr_2_ (#1)^11^ and Fe_35_Mn_45_Co_10_Cr_10_ (#3) alloys upon tensile deformation at room temperature. Interestingly, the homogenized single-phase non-equiatomic Fe_40_Mn_40_Co_10_Cr_10_ alloy (#4) shows significantly higher ultimate strength (~95 MPa) compared with the equiatomic alloy (#2) due to the introduction of a higher extent of twinning behavior.^12^ Along with the alloy design strategies explained above, the newly designed quinary TRIP-assisted dual-phase Co_20_Cr_20_Fe_34_Mn_20_Ni_6_ alloy (#5)^23^ has further enhanced ultimate strength compared with the TWIP-assisted Fe_40_Mn_40_Co_10_Cr_10_ alloy (#4).

Furthermore, the homogenized dual-phase non-equiatomic Fe_50_Mn_30_Co_10_Cr_10_ alloy (Fig. 6; #6) shows much higher ultimate strength compared with the various single-phase alloys (#1–4) and the dual-phase Co_20_Cr_20_Fe_34_Mn_20_Ni_6_ alloy (#5), while maintaining total elongation above 50%. Interestingly, the alloy with the same composition but refined FCC matrix grains (#7) exhibits further significant joint increase of strength and ductility. This is ascribed to the substantially improved work-hardening ability of the alloy due to the well-tuned phase stability via adjustment of grain size and phase fractions.^4^,^13^ With addition of interstitial element carbon into the dual-phase microstructure, the grain-refined Fe_49.5_Mn_30_Co_10_Cr_10_C_0.5_ alloy (#8) shows further increased ultimate strength up to nearly 1 GPa with total elongation of ~60%. These superior mechanical properties are attributed to the joint activity of various strengthening mechanisms including interstitial and substitutional solid solution, TWIP, TRIP, nanoprecipitates, dislocation interactions, stacking faults, and grain boundaries.^25^

To further clarify the mechanisms responsible for the above microstructure–property relations, Fig. 7 provides an overview of the various deformation mechanisms in different multicomponent HEAs presented in Fig. 6. From the single-phase Fe_40_Mn_27_Ni_26_Co_5_Cr_2_ and Fe_35_Mn_45_Co_10_Cr_10_ alloys towards the single-phase Co_20_Cr_20_Fe_20_Mn_20_Ni_20_ and Fe_40_Mn_40_Co_10_Cr_10_ alloys, a TWIP effect has been introduced. Then, the presence of phase boundaries (dual-phase structure) and the TRIP effect are included in the dual-phase Co_20_Cr_20_Fe_34_Mn_20_Ni_6_ and Fe_50_Mn_30_Co_10_Cr_10_ alloys. Proceeding further to the carbon-containing Fe_49.5_Mn_30_Co_10_Cr_10_C_0.5_ alloy, interstitial solid solution and nanoprecipitate strengthening effects are additionally utilized, thereby unifying all known strengthening mechanisms in one material. Indeed, these multiple deformation mechanisms enable significant improvement of the strain-hardening capacity and strength–ductility combination. This clearly shows that tuning deformation mechanisms via composition adjustment is key to the design of strong and ductile non-equiatomic HEAs. Therefore, we suggest that introduction of multiple deformation mechanisms which are activated gradually or, respectively, sequentially during mechanical and/or thermal loading is a key route for future development of new HEAs with advanced mechanical properties. Such a concept can be realized by shifting from single-phase equiatomic to dual- or multiphase non-equiatomic compositional configurations.

## Summary and Outlook

We have presented a brief overview on the design, processing, microstructure, and mechanical properties of non-equiatomic HEAs. As the most widely studied HEA family, we mainly focused on 3*d* transition-metal HEAs, which exhibit a broad spectrum of microstructures and mechanical behavior. We showed that various strong and ductile HEAs can be obtained by shifting the alloy design strategy from single-phase equiatomic to dual- or multiphase non-equiatomic compositions. The non-equiatomic HEA concept provides possibilities for the unification of various strengthening and toughening mechanisms, enabling significant improvement of strain-hardening capacity and strength–ductility combinations. To design strong and ductile non-equiatomic transition-metal HEAs, the intrinsic stacking fault energies and/or the free energy differences between the FCC and HCP phases for various compositions can be probed in an effort to tune the phase stabilities by adjusting the compositions. Proper processing routes toward homogeneously distributed multiple principal elements and appropriate grain sizes are also important for achieving targeted strength–ductility combinations, especially for dual-phase non-equiatomic HEAs with TRIP effect.

Although outstanding strength and ductility have been achieved in several non-equiatomic transition-metal HEAs, e.g., ultimate tensile strength of ~1 GPa and total elongation of ~60% in the above-mentioned TWIP-TRIP-iHEA at room temperature, further research into strong and ductile non-equiatomic HEAs is still required; possible future trends include:

1. The strength and ductility of the various non-equiatomic HEAs at low and elevated temperatures are still unknown, and new (non-equiatomic) HEAs with excellent strength–ductility combinations at low and elevated temperatures can be designed and studied.
2. For the widely studied transition-metal HEAs with their good strength–ductility combinations, other properties such as the resistance to hydrogen-induced degradation,^28^,^29^ corrosion resistance, fatigue behavior, and magnetic performance can also be explored in an effort to find superior combinations of properties, i.e., multifunctionalities, to justify their relatively high cost compared with established high-strength and austenitic stainless steels. Also, following the design approach associated with the stacking fault energies and/or the free energies, a shape-memory effect could be introduced into non-equiatomic HEAs.
3. Other than the transition-metal family, non-equiatomic HEAs containing refractory elements such as Ti, Nb, Ta, Zr, Hf, V, Mo, and W^30^ can also be developed towards high-performance refractory HEAs as high-temperature load-bearing structures for application in the aerospace industry.
4. Considerable research work can also be conducted to screen the effects associated with different minor interstitial element fractions (C, N, B, O, etc.) to further improve the performance of the various non-equiatomic HEAs and understand the corresponding mechanisms.
